# High-rate squeezing process of bulk metallic glasses

**DOI:** 10.1038/srep45051

**Published:** 2017-03-24

**Authors:** Jitang Fan

**Affiliations:** 1 State Key Laboratory of Explosion Science and Technology, Beijing Institute of Technology, Beijing 100081, China; 2 Advanced Research Institute for Multidisciplinary Science, Beijing Institute of Technology, Beijing 100081, China

## Abstract

High-rate squeezing process of bulk metallic glasses from a cylinder into an intact sheet achieved by impact loading is investigated. Such a large deformation is caused by plastic flow, accompanied with geometrical confinement, shear banding/slipping, thermo softening, melting and joining. Temperature rise during the high-rate squeezing process makes a main effect. The inherent mechanisms are illustrated. Like high-pressure torsion (HPT), equal channel angular pressing (ECAP) and surface mechanical attrition treatments (SMAT) for refining grain of metals, High-Rate Squeezing (HRS), as a multiple-functions technique, not only creates a new road of processing metallic glasses and other metallic alloys for developing advanced materials, but also directs a novel technology of processing, grain refining, coating, welding and so on for treating materials.

The discovery of metallic glassy materials with a disordered atomic structure is the advent of a new class of advanced materials, which have a unique combination of outperforming mechanical and physical properties for a great potential in various applications^1^,^2^,^3^,^4^. Shear banding always operates the deformation and fracture process of bulk metallic glasses, when subjected to an applied stress^5^,^6^,^7^,^8^. This mechanism contributes to the plastic straining. Accompanying with it, temperature rise occurs. Due to the ‘hot’ shear bands, strain softening and melting behaviour are induced in the deformation and fracture process^9^,^10^,^11^,^12^.

A number of publications have reported the phenomenon of temperature-rise-induced melting behaviour during a ‘hot’ shear banding in metallic glasses^9^,^10^,^11^. By using a fusible coating, Lewandowski *et al*.^10^ estimated that temperature rise can approach a few thousand kelvin during shear banding in metallic glasses. It is thought to result in the material melting inside shear bands, and thus leading to prominent vein-like structures and liquid droplets on the fracture surface. Also, a melting liquid layer on the fracture surface of metallic glasses was reported, which was evaluated to have several micrometers thickness^7^.

On another hand, Wright *et al*.^13^,^14^ believed that the final fracture processing, rather than the shear banding, results in the melting behaviour of metallic glasses by evaluating the temperature rise of more than 209 kelvin in a shear band. An unstable shear banding, even accompanied with a low temperature rise, can finally evolve into a hot fracturing, and thus resulting in the melting behaviour^15^.

From above, we can conclude that temperature rise and resultant melting behaviour in metallic glasses caused by deformation and fracture process has a broad consensus^9^,^10^,^11^,^12^,^13^,^14^,^15^. Utilizing this unique performance^16^,^17^,^18^, processing bulk metallic glasses is provided an applicable potential at room temperature, as well as other applications.

In this study, we design a high-rate squeezing (HRS) technique to realize the processing of bulk metallic glasses at room temperature from a cylinder into an intact sheet. It opens a new road of processing various metallic glasses and other metals, and establishes a novel applicable technique with multiple functions and applications.

## Experimental Methods

In this research, the bulk metallic glasses is selected as Zr_52.5_Cu_17.9_Ni_14.6_Al_10_Ti_5_ (Vitreloy 105) with a fully amorphous microstructure, which has a high glass forming ability (GFA) and a good combination of strength and toughness^19^. The used specimens of 2 mm-diameter and 2.4 mm-height cylindrical rod were prepared by arc melting a mixture of the pure metallic components under a Ti-gettered argon atmosphere followed by suction cast into a water-cooled Cu mold. The HRS technique is performed by an apparatus of split Hopkinson pressure bar (SHPB), which has been widely used to characterize the dynamic mechanical response of various engineering materials deforming at high strain rates (10^2^–10^4^/s)^20^,^21^,^22^,^23^. Herein, the applied SHPB has a diameter of 14.5 mm and is made by the material of 18Ni high-strength steel with yield strength of about 2000 MPa, which ensures a fully persisted elastic deformation during the HRS processing of metallic glassy specimens (yield strength of 1800 MPa). The processing work is conducted at room temperature, which exerts a high-rate loading on the specimen of cylindrical rod. The processing rates from 400–15000/s were operated and the related stress-strain curves were derived.

In a SHPB test, the well-established elementary linear elastic wave propagation theory can be employed to calculate the stress, strain and strain-rate versus time relations, based on the recorded strains of incident bar and transmitter bar^22^,^23^, as 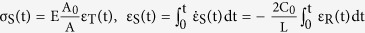 and 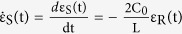. Wherein, σ_s_(t), ε_s_(t) and 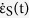 are the specimen’s stress, strain and strain rate, respectively; E is the bar’s Young’s modulus; A_0_ is the bar’s cross sectional area; A is the specimen’s initial cross sectional area of gauge section, i.e. deformation part of the specimen; ε_T_(t) is the transmitted strain history; ε_R_(t) is the reflected strain history; L is the initial gauge length of the specimen and C_0_ is the wave velocity in the incident bar, calculated as 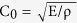, in which ρ is the bar density.

## Results and Discussions

**Figure 1:**
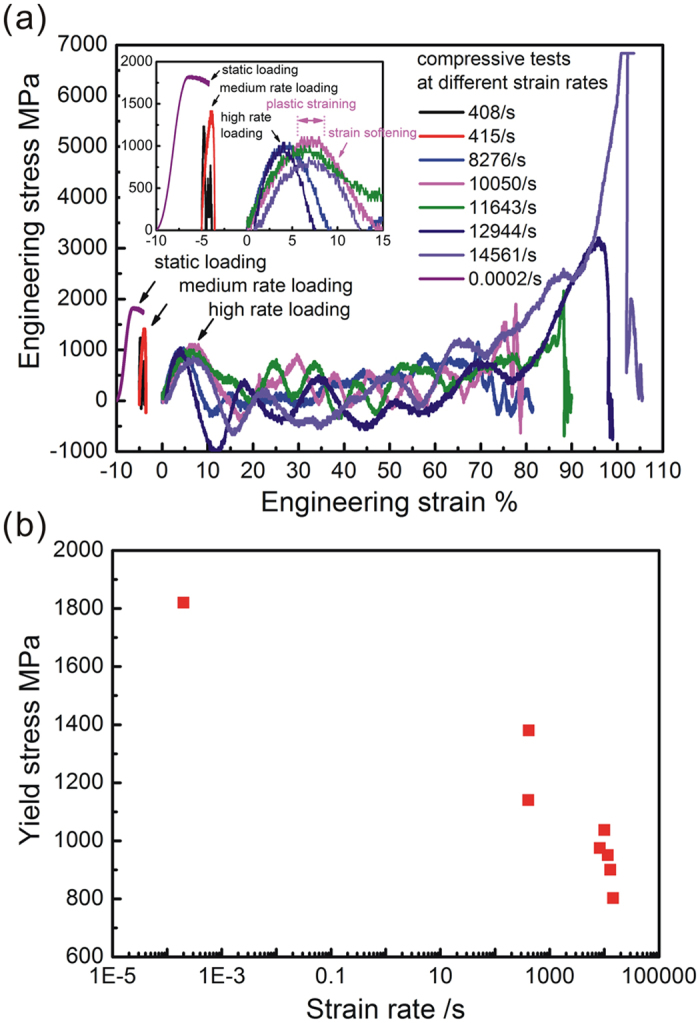
Rate-dependent mechanical properties of Vitreloy 105 metallic glasses: (**a**) uniaxial compressive stress-strain curves at various loading rates; (**b**) rate dependency of yield stress.

Figure 1 shows the rate-dependent mechanical properties of Vitreloy 105 metallic glasses. Under static loading, Vitreloy 105 metallic glasses displays a yielding at 1800 MPa, followed by a plastic straining of 2.6% up to the final fracture (see Fig. 1(a)). Under medium rate loading of about 400/s, yield stress approximates to 1300 MPa, almost without any plastic straining before the final fracture.

Under high rate loading of more than 10000/s, yield stress decreases down to lower than 1000 MPa. However, it is interesting to note that plastic strain of 2–3% occurs followed by a strain softening (see the inset of Fig. 1(a)). Afterwards, local fracture may occur to the specimen, resulting in a sudden stress drop. Due to the inertia of pressure bar, such a sudden stress drop leads to the reflection of elastic wave, which forms a tensile stress in the pressure bar. This phenomenon indicates the effect of pressure bar stiffness on deformation behaviour of metallic glasses^24^,^25^. Then, because of the geometric confinement for the deformed specimen (height-to-diameter ratio is about 1.2) induced by the pressure bar, catastrophic fracture is restrained. Instead, a large plastic flow is triggered up to about 80% engineering strain at a low stress level. Accompanying with it, multiple shear bandings/slippings will be induced^24^,^26^, which results in the temperature rise. So, thermo softening is formed to cause a low flowing stress^11^. Meanwhile, melting behaviour of metallic glassy material may occur. Finally, the deformed specimen is squeezed into a thin sheet, during which the engineering stress is sharply increased, as shown in the part of stress-strain curve of more than 80% engineering strain. Herein, strain rate is calculated from the initial straining before squeezing, which is defined as processing rate. For the HRS process, squeezing rate can be calculated as ΔL/t. Herein, ΔL is the specimen length difference before and after squeezing, and t is the loading time, which is equal to the duration time of incident wave. The calculation data will be illustrated later.

Rate dependency of yield stress is illustrated in Fig. 1(b), which displays a decreasing tendency with the increase of strain rate. A softening behaviour induced by loading rate can be imaged^27^,^28^,^29^,^30^, which indicates more temperature rise at higher strain rate. Thus, the resultant melting behaviour of metallic glassy material is expected to be evolved with the strain rate.

**Figure 2:**
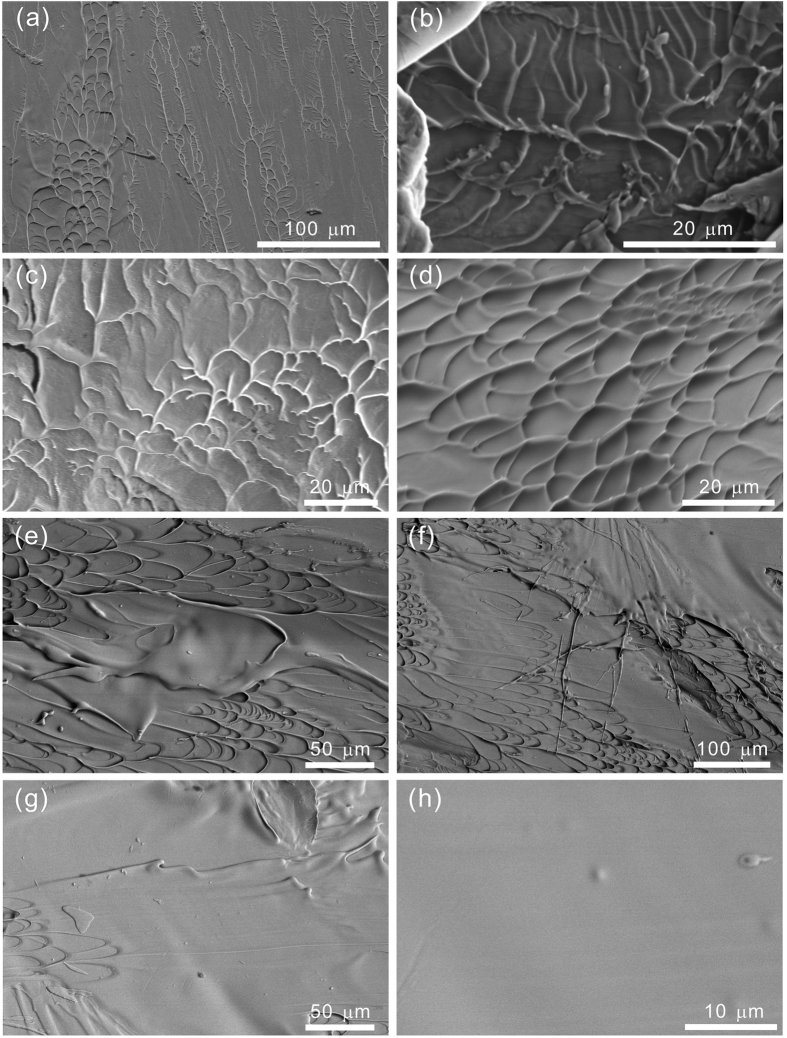
Melting behaviour evolved with loading rate revealed by observing fracture surface patterns. Under static loading: (**a**) a joint formation of dense vein-like structures and dendrite-like patterns; Under medium rate loading: (**b**) dendrite-like patterns and (**c**) sparse vein-like structures; Under high rate loading: (**d**) dense vein-like structures with extended characteristics, (**e**) dense vein-like structures and melting liquid, (**f**) a thick layer of melting liquid covering the vein-like structures, (**g**) fully melting liquid and (**h**) featureless melting liquid with a smooth surface.

Figure 2 reveals the melting behaviour of Vitreloy 105 metallic glasses evolved with loading rate by observing the patterns on fracture surface. Under static loading, both the dense vein-like structures and dendrite-like patterns are observed on the fracture surface (see Fig. 2(a)), which corresponds to the plastic deformation (see Fig. 1(a)). Under medium rate loading, dendrite-like patterns are mainly formed (see Fig. 2(b)) and vein-like structures become sparse (see Fig. 2(c)). It is in line with the observations on fracture surface of metallic glasses after dynamic loading^31^. Thus, the decreased yield stress and absence of plasticity are reasonable for the “hot” shear banding and fast shearing fracture droved by dynamic stress. Under high rate loading, the dense vein-like structures with extended characteristics occur overwhelmingly (see Fig. 2(d)), accompanied with the melting liquid (see Fig. 2(e)), which indicates the viscous flow. It corresponds to the appearance of plastic deformation (see the inset of Fig. 1(a)).

Furthermore, a thick layer of melting liquid is found covering the vein-like structures (see Fig. 2(f)), which indicates a higher temperature rise. The higher temperature rise leads to more softening behaviour, contributing to a further strength decrease (see Fig. 1), and results in profuse melting behaviour endowing a viscous fluidity^3^,^9^,^10^. Temperature rise in metallic glassy materials was extensively investigated by a coupled thermo-mechanical model^32^,^33^. They predicted that it is the results of shear band evolution and strongly depends on the proportionality coefficient and the local strain rate^32^. Shear banding can result in a significant temperature rise in ductile Zr-based metallic glasses, otherwise cracking may take place and cause the catastrophic fracture, like the case in brittle Fe- and Mg-based metallic glasses. The flow stress depends on the temperature as well as the sliding speed of shear band^33^. An increased temperature can lower the shear resistance in shear band and therefore facilitates the sliding which in turn supplies the heating energy into the band to further elevate the temperature.

In the current case, a thick layer of melting liquid is formed in Vitreloy 105 (Zr-based) metallic glasses at a high strain rate. Inside this layer, the metallic glassy material is fully melted. Matthews^34^ *et al*. conducted both TEM and SEM observations to experimentally confirm the presence of a layer of melting liquid on the fracture surface of a metallic glassy material. Moreover, by both experimental evaluation and model calculation, they concluded that the thickness of the layer of melting liquid can substantially exceed the generally accepted thickness of a shear band. Thus, the resultant dramatic decrease of viscosity at a high strain rate and high proportionality coefficient indicates that a significant strain rate softening would occur in the final stage of shear band evolution^32^. So, the current Vitreloy 105 metallic glasses shows a lower yield stress and a certain plastic flow under high rate loading. Even, a thicker layer of fully melting liquid is formed under a higher rate loading (see Fig. 2(g)). It is featureless with a smooth surface (see Fig. 2(h)), which, on the one hand, prevents the shear bands growing into cracks, on the other hand, heals the micro cracks. The average thickness of melting layer is supposed to increase with the increasing strain rate, which is strain rate dependent.

By comparing the patterns of fracture surface as shown in Fig. 2(c–h), we can conclude that the smoothness of fracture surface becomes higher with the increasing strain rate. The reason is analyzed that with the increasing strain rate, temperature rise becomes higher, and the resultant melting liquid is more, which causes a lower speed of heat conduction and a slower cooling rate. Surface energy of melting liquid has enough time to smooth the fracture surface. So, we can summarize that fracture surface becomes smoother with the increasing strain rate, because of a lower cooling rate at higher strain rate. Fracture surface smoothness is also strain rate dependent.

From above illustrations, we can conclude that under high rate loading (more than 10000/s strain rate), temperature rise is high enough to result in a fully melting behaviour inside a thick layer of Vitreloy 105 metallic glassy material. Such the high temperature rise and resultant melting behaviour under a high rate loading contribute to the HRS processing technique at room temperature. It is one of the intrinsic characteristics of high-rate squeezing (HRS) technique.

**Figure 3:**
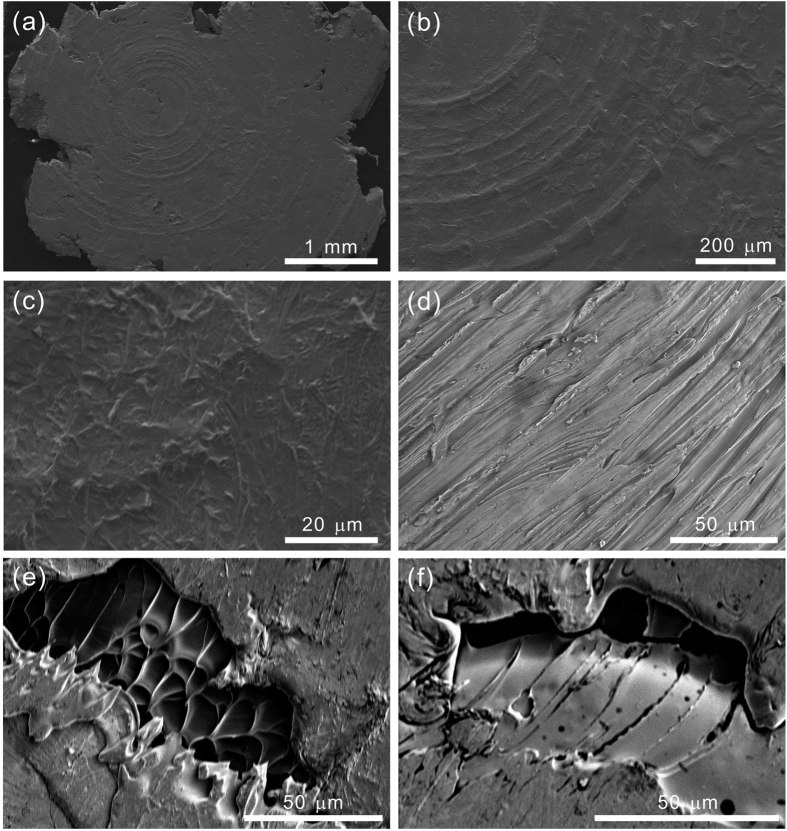
Processing of Vitreloy 105 metallic glasses by high-rate squeezing (HRS) technique at room temperature: (**a**) an approximate 4 mm-diameter and 0.3 mm-thickness sheet formed from a cylinder coating on a high-strength steel substrate; (**b**) circular stripes on the top surface of the sheet induced by a corresponding HRS mould; (**c**) intact sheet without observing any damage; (**d**) patterns formed by the extended and scraped melting liquid; (**e**) dense vein-like structures and (**f**) wiped melting liquid underneath the top surface of the sheet.

Figure 3 shows the post-process patterns of Vitreloy 105 metallic glasses conducted by high-rate squeezing (HRS) technique at room temperature. An approximate 4 mm-diameter and 0.3 mm-thickness sheet is produced from a cylinder after high-rate squeezing (see Fig. 3(a)), which coats on a high-strength steel substrate. Thus, the squeezing rate can be calculated as 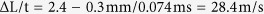. Herein, 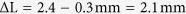, which is the specimen height difference before and after squeezing; and, t is the loading time, which is equal to the duration time of incident wave, i.e. 0.074 ms in the current SHPB test. Several circular stripes are formed on the top surface of the squeezed sheet induced by a corresponding HRS mould (see Fig. 3(b)), which indicates that the surface patterns can be modified by a determined HRS mould. An amplified image reveals that the squeezed sheet is intact without any damage and the surface is rough (see Fig. 3(c)). The melting liquid is extended during high-rate squeezing and is scraped by the rough surface of the HRS mould (see Fig. 3(d)). These observations indicate that temperature rise during HRS process is high enough to melt the surface of metallic glassy material^10^. The dense vein-like structures (see Fig. 3(e)) and the wiped melting liquid (see Fig. 3(f)) are found underneath the top surface of the sheet, which further confirm the temperature rise and the resultant melting behaviour accompanying with the HRS process. Furthermore, these micro cracks are expected to be restrained of growing into macro cracks or to be healed from macro cracks by the melting liquid. Therefore, a large-size undamaged thin sheet can be produced by high-rate squeezing (HRS) technique at room temperature. The roughness and pattern of the sheet surface can be modified by HRS mould. This performance can contribute to a flexible processing technology and a sound coating/welding behaviour.

**Figure 4:**
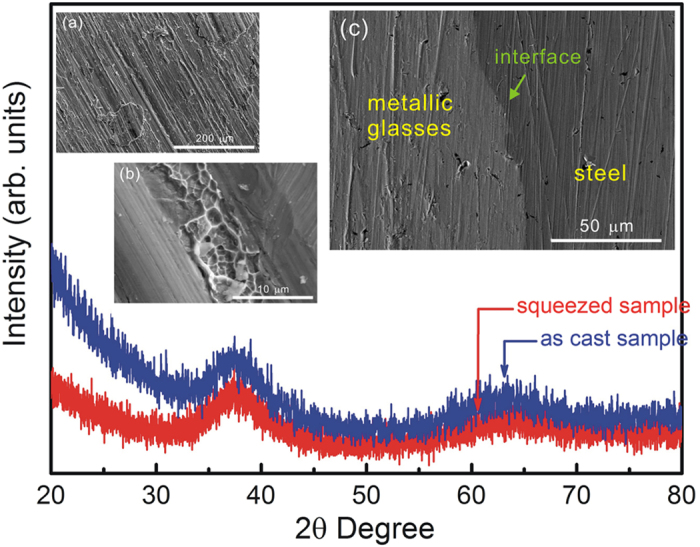
XRD patterns revealing the amorphous microstructure of Vitreloy 105 metallic glasses after HRS process and SEM images showing the welding behaviour: (**a**) rough surface of the squeezed sheet with numerous parallel ridges; (**b**) vein-like structures between the ridges; (**c**) welding behaviour between the metallic glasses and high-strength steel with an intact interface.

XRD patterns reveal the amorphous microstructure of the squeezed sheet (see Fig. 4), which means that the intrinsic microstructure of metallic glasses is not changed by the melting behaviour during high-rate squeezing (HRS) process. It is attributed to the high glass forming ability of Vitreloy 105 metallic glasses^19^. The insets reveal the rough surface of the squeezed sheet with numerous parallel ridges (see Fig. 4(a)), and the vein-like structures between the ridges (see Fig. 4(b)), which are significant for the welding behaviour by a physical or/and mechanical bonding^35^. The welding behaviour between the metallic glasses and high-strength steel is realized by high-rate squeezing (HRS) technique (see Fig. 4(c)). The interface is intact and is expected to form a solid bonding, considering the high-temperature and high-pressure process in HRS technique. Later on, mechanical strength, plasticity, hardness and other physical performance of the squeezed sheet will be investigated. Bonding stress as well as bonding mechanisms of metallic glasses and high-strength steels produced by HRS process will also be the research interests in science and technology.

**Figure 5:**
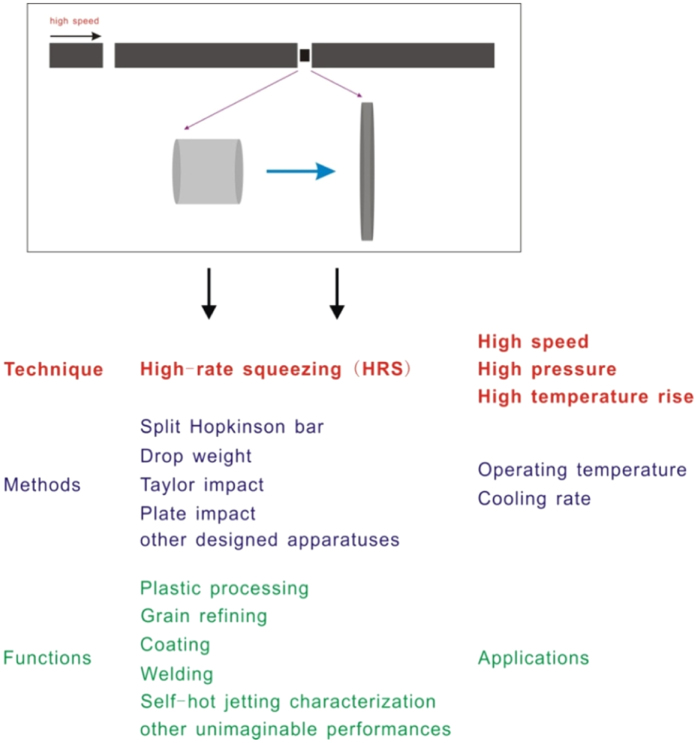
High-rate squeezing (HRS) technique: inherent technological mechanisms; processing methods and operating environments; and achieved functions and applications.

To extend, high-rate squeezing (HRS) is a comprehensive material processing technique with the characteristics of high speed, high pressure and high temperature rise, whose inherent technological mechanisms, processing methods and operating environments, as well as the achieved functions and applications are listed in Fig. 5. Besides split Hopkinson bar, the processing apparatus can be drop weigh, Taylor impact, plate impact, explosive and other designed equipments that can produce a high-rate compressive loading^23^,^35^. These apparatuses are well-used techniques with quantificational working parameters, which can ensure the reliability and repeatability of the HRS processing results. The operating environments can be at different processing temperatures and in various working mediums for varying the cooling rate after HRS process.

HRS is a processing technique conducted by various high-rate compressive apparatuses in a variable working environment. The HRSed materials can include the metallic glasses and their composites, high-strength metallic alloys, non-equilibrium alloys and various coarse-grain alloys. The processing results are multiple and colorful. Coarse grain can be refined into ultrafine and nano grain, like the techniques of high-pressure torsion (HPT)^36^, equal channel angular pressing (ECAP)^37^ and surface mechanical attrition treatments (SMAT)^38^. Some metallic glasses can be induced a partial nanocrystallization^39^. The resultant sheet-shape alloys by such the severe deformation of HRS technique can be a monolith or mixture of amorphous, nano, ultrafine, and coarse grains, that are expected to have the unique mechanical and physical properties.

In HRS technique, welding behaviour^35^ can be produced by placing a medium in-between two welded alloys, where the medium can be metallic glasses or other metallic alloys as the solder. Furthermore, coating behaviour can also be produced via achieving the welding behaviour in a single side. The interface is expected to be intact, to form a high-strength bonding and to have other high-performance physical properties. Herein, due to the instantaneous process of HRS technique (much less than 1 ms) at room temperature, interface oxidation effect can be almost ruled out^40^,^41^, which contributes to a well bonding condition at interface. It is advantageous comparing with the high temperature joining technique.

During HRS processing, bulk metallic glasses shows a high strength of more than 800 MPa, which is comparable with engineering steel, and melting behaviour is induced due to temperature rise. Such the performance is characterized as the hot jetting in the penetrating process of a penetrator^42^. So, a self-hot jetting function of metallic glasses can be revealed by the HRS technique. Metallic glasses and their composites are supposed to be coated on a penetrator for improving the penetration capability.

Therefore, high-rate squeezing (HRS) technique with the characteristics of high speed, high pressure and high temperature rise is established, which can process a variety of metallic alloys via various high-rate compressive apparatuses for achieving multiple functions and applications.

## Conclusions

To conclusion, high-rate squeezing performance of bulk metallic glasses is investigated, operated by split Hopkinson pressure bar (SHPB), which produces a large-size intact thin metallic glassy sheet from a cylindrical specimen. It is a technique progressing of processing bulk metallic glasses, which is comparable with the nanoscale imprinting of metallic glassy materials^43^,^44^. Inherent mechanism of such a considerable shape change is the instantaneous plastic flow of metallic glassy material, which is involved in the geometrical confinement, shear banding/slipping, thermo softening, melting and joining into a thin sheet. The intact thin sheet is expected to have a sound coating/welding behaviour with high-strength steel revealed by an undamaged, rough interface. Based on this study, high-rate squeezing (HRS) technique is firstly proposed, which is a processing method with extensively applicable apparatuses and operating environments for treating various metallic materials to produce an intended result.

## Additional Information

**How to cite this article:** Fan, J. T. High-rate squeezing process of bulk metallic glasses. *Sci. Rep.* **7**, 45051; doi: 10.1038/srep45051 (2017).

**Publisher's note:** Springer Nature remains neutral with regard to jurisdictional claims in published maps and institutional affiliations.
